# High mobility Ge pMOSFETs with amorphous Si passivation: impact of surface orientation

**DOI:** 10.1186/s11671-018-2847-0

**Published:** 2019-01-08

**Authors:** Huan Liu, Genquan Han, Yan Liu, Xiaosheng Tang, Jingchen Yang, Yue Hao

**Affiliations:** 1 State Key Discipline Laboratory of Wide Band Gap Semiconductor Technology, School of Microelectronics, Xidian University, Xi’an, 710071 China; 2 College of Optoelectronic Engineering, Chongqing University, Chongqing, 400044 China

**Keywords:** Germanium, MOSFET, Amorphous Si passivation, Mobility, Surface orientation

## Abstract

We report the amorphous Si passivation of Ge pMOSFETs fabricated on (001)-, (011)-, and (111)-orientated surfaces for advanced CMOS and thin film transistor applications. Amorphous Si passivation of Ge is carried out by magnetron sputtering at room temperature. With the fixed thickness of Si *t*_Si_, (001)-oriented Ge pMOSFETs achieve the higher on-state current *I*_ON_ and effective hole mobility *μ*_eff_ compared to the devices on other orientations. At an inversion charge density *Q*_inv_ of 3.5 × 10^12^ cm^−2^, Ge(001) transistors with 0.9 nm *t*_Si_ demonstrate a peak *μ*_eff_ of 278 cm^2^/V × s, which is 2.97 times higher than the Si universal mobility. With the decreasing of *t*_Si_, *I*_ON_ of Ge transistors increases due to the reduction of capacitive effective thickness, but subthreshold swing and leakage floor characteristics are degraded attributed to the increasing of midgap *D*_it_.

## Background

Germanium (Ge) has been attracting tremendous research interests for advanced CMOS and thin film transistor applications due to its higher hole mobility and lower thermal budget processing compared to Si [1–6]. To achieve the high channel mobility, the surface passivation process leading to a high interface quality is required before gate stack formation. Several surface passivation techniques have been developed to deliver the carrier mobility benefits in Ge metal-oxide-semiconductor field-effect transistors (MOSFETs) [1, 2, 7–10]. Among these techniques, a silicon (Si) cap passivated on Ge has been the hotspot in recent years, due to its advantages of effective suppressing of interface states and good thermal stability and reliability [11]. Formation of Si passivation cap has been widely studied using chemical vapor deposition (CVD) with precursors of SiH_4_ [1], Si_2_H_6_ [4], Si_3_H_8_ [12], and E-beam evaporation [13]. Although CVD method could provide the more uniform passivation layer over physical vapor deposition (PVD), its passivation rate has the strong correlation in channel surface orientation and the process temperature. PVD technique could provide the improved passivation rate even at room temperature, which has the advantages of low thermal budget and low cost, making it more suitable for the thin film transistors and back-end-of-line 3D integration applications. In this letter, we fabricated high mobility Ge pMOSFETs on (001)-, (011)-, and (111)-oriented surfaces utilizing amorphous Si passivation by magnetron sputtering. Significantly improved effective hole mobility *μ*_eff_ is achieved in Ge transistors compared to the Si universal mobility. Impacts of surface orientation and thickness of amorphous Si *t*_Si_ on the boosting effect of amorphous Si passivation on *μ*_eff_ are studied.

## Methods

**Fig. 1:**
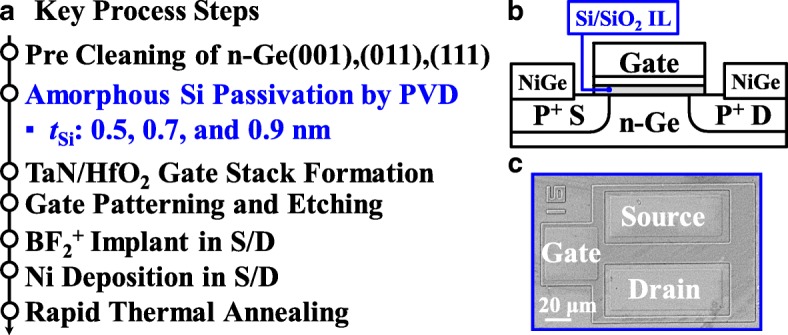
**a** Process sequence showing the key steps employed to fabricate the Ge pMOSFETs with different *t*_Si_. **b** Cross-sectional schematic of a Ge pMOSFET with SiO_2_ IL. **c** Top-view microscope image of a fabricated Ge pMOSFET

Figure 1a shows the key process steps for fabricating Ge pMOSFETs on (001)-, (011)-, and (111)-oriented surfaces. After pre-gate cleaning in diluted HF (1:50) solution, ultrathin amorphous Si passivation layer was deposited on n-Ge substrates by magnetron sputtering at a target power of 50 W. Three passivation durations of 60 s, 80 s, and 100 s were used corresponding to the deposition of 0.5, 0.7, and 0.9 nm *t*_si_, respectively. After that, a 5-nm thick HfO_2_ gate dielectric was deposited at 250 °C by atomic layer deposition using TDMAHf and H_2_O as precursors of Hf and O, respectively. A 50-nm TaN gate electrode was deposited by reactive sputtering. Next, the gate electrode was patterned and etched, which was followed by BF_2_^+^ implantation into source/drain (S/D) regions at 30 KeV with a dose of 1 × 10^15^ cm^− 2^. Non-self-aligned S/D metals of 15-nm nickel were formed by lift-off process. Finally, rapid thermal annealing at 400 °C was carried out for dopant activation and S/D metallization. Figure 1b shows the cross-sectional schematic of the Ge pMOSFET with Si/SiO_2_ interfacial layer (IL). Figure 1c shows top-view microscope image of a fabricated Ge pMOSFET.

**Fig. 2:**
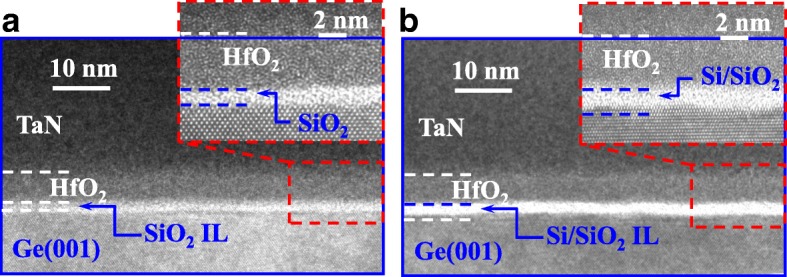
Cross-sectional TEM images of Ge pMOSFET gate stacks with **a** 0.5 nm *t*_Si_ and **b** 0.9 nm *t*_Si_. HRTEM images in insets show that Si/SiO_2_ IL is formed between HfO_2_ and Ge channel

Figure 2a, b shows the transmission electron microscope (TEM) images of the high-κ/metal gate stack with SiO_2_/Si interfacial layer (IL) on Ge(001) channel with *t*_Si_ of 0.5 and 0.9 nm, respectively. Insets show the high-resolution TEM (HRTEM) images of the samples. For the device with a *t*_Si_ of 0.5 nm, amorphous Si layer was completely oxidized, while for the device with 0.9 nm *t*_Si_, about two Si monolayers remained after the subsequent annealing steps.

## Results and discussion

**Fig. 3:**
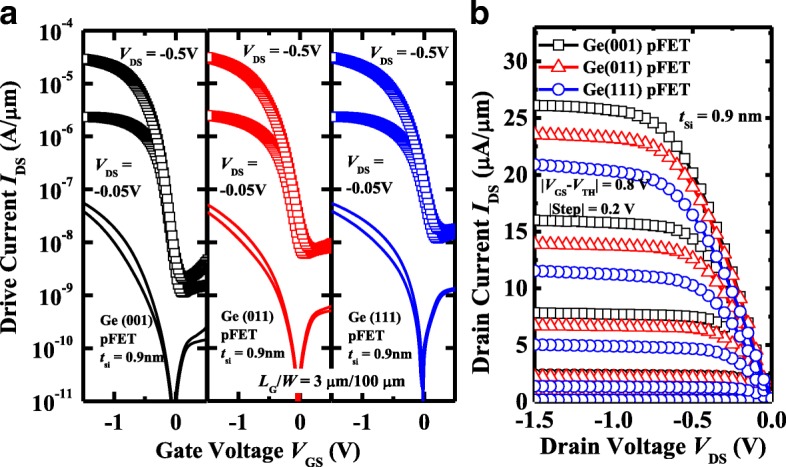
**a** Measured *I*_DS_-*V*_GS_ and *I*_G_-*V*_GS_ curves of (001)-, (011)-, and (111)-oriented Ge pMOSFETs with 0.9 nm *t*_Si_ showing the excellent transfer characteristics. **b** *I*_DS_-*V*_DS_ curves measured at different *V*_GS_-*V*_TH_ for the devices

**Fig. 4:**
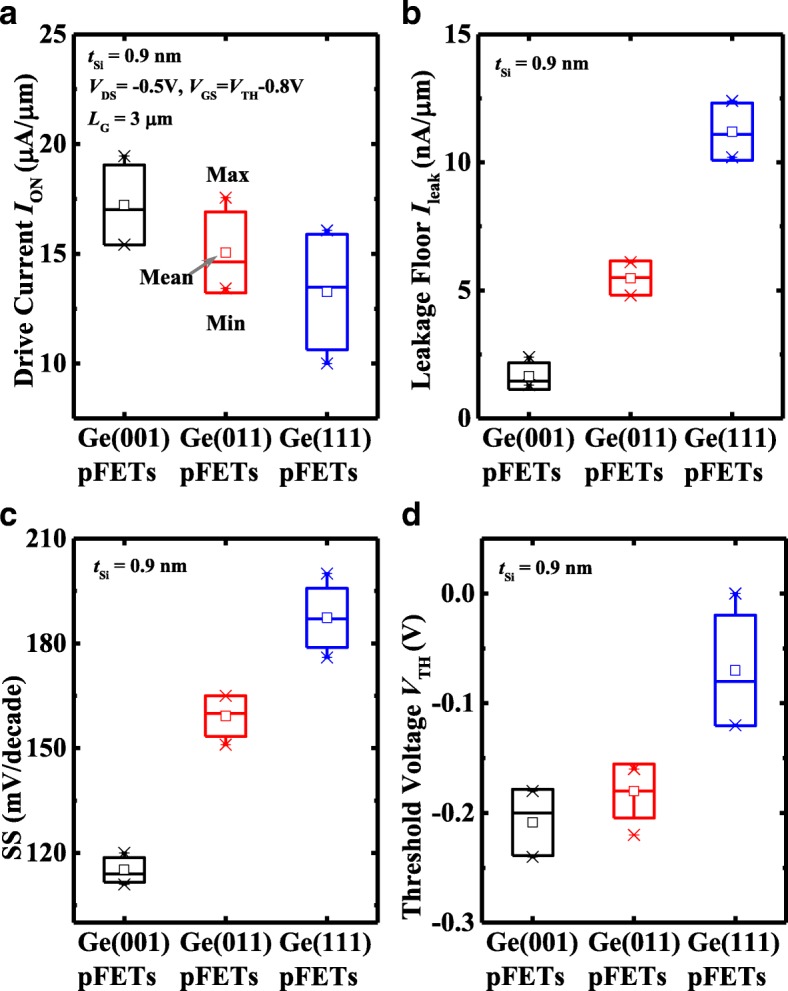
Comparison of **a** *I*_ON_, **b** *I*_leak_, **c** SS, and **d** *V*_TH_ for (001)-, (011)-, and (111)-oriented Ge pMOSFETs with a *t*_Si_ of 0.9 nm

Figure 3a plots the measured *I*_DS_-*V*_GS_ and *I*_G_-*V*_GS_ curves of the typical Ge pMOSFETs on (001)-, (011)-, and (111)-oriented surfaces with 0.9 nm *t*_Si_, which show the excellent transfer characteristics. All transistors have a gate length *L*_G_ of 3 μm and a gate width *W* of 100 μm. The channel direction is [110] for all the orientations. The *I*_DS_-*V*_DS_ curves of the devices measured at different gate overdrive *V*_GS_-*V*_TH_ are shown in Fig. 3b. Here, threshold voltage *V*_TH_ is defined as the *V*_GS_ at *I*_DS_ of 10^−7^ A/μm. It is observed that Ge(001) pMOSFET achieves the higher drive current *I*_ON_ compared to the transistors on (011) and (111) surfaces at the fixed *V*_GS_-*V*_TH_. Later, we will show that this is attributed to the fact that Ge(001) pMOSFETs have a higher effective hole mobility *μ*_eff_ in comparison with the devices on the other two surface orientations. We perform a comprehensive comparison of electrical performance for the devices with the fixed *t*_Si_ of 0.9 nm, including *I*_ON_, leakage floor *I*_leak_, subthreshold swing (SS), and *V*_TH_ characteristics. *I*_leak_ is defined as the minimum *I*_DS_ at *V*_DS_ of − 0.05 V. Figure 4a presents the statistical plot of the *I*_ON_ for Ge pMOSFETs on various orientations, and *I*_ON_ was defined as *I*_DS_ at a *V*_DS_ of − 0.5 V and a *V*_GS_-*V*_TH_ of − 0.8 V. All the transistors in this plot have the *L*_G_ of 3 μm and *W* of 100 μm. (001)-oriented devices exhibit the improved mean *I*_ON_ as compared to those on (011) and (111) orientations, which is attributed to the higher *μ*_eff_. Figure 4b compares the *I*_leak_ for the devices, showing that Ge(001) transistors have the lowest *I*_leak_ of them, and Ge(011) pMOSFETs have the lower *I*_leak_ than (111)-oriented devices. It should be noted that the *I*_leak_ is determined by the reverse current of the p^+^/n junction in drain region, which is affected by the background n-type doping concentration in Ge substrate and activation of the implanted p^+^ dopants. The n-type doping concentrations in the wafers with various orientations are not exactly the same. The surface orientation affects the dopant activation rate and recrystallization quality of S/D regions. Furthermore, although the *I*_G_ is lower than *I*_DS_ before the turn-on of the transistors, it would influence the *I*_leak_. Similarly, (001)-oriented Ge pMOSFETs demonstrate the improved SS characteristics in comparison with other two orientations, which is due to that transistors on (001) surface have the lower midgap density of interface state *D*_it_ compared to the other devices. Figure 4d shows that the devices on different orientations have the different *V*_TH_. Based on the results in Fig. 4, it is concluded that, with the fixed *t*_Si_ of 0.9 nm, (001)-oriented Ge pMOSFETs obtain the best electrical characteristics.

**Fig. 5:**
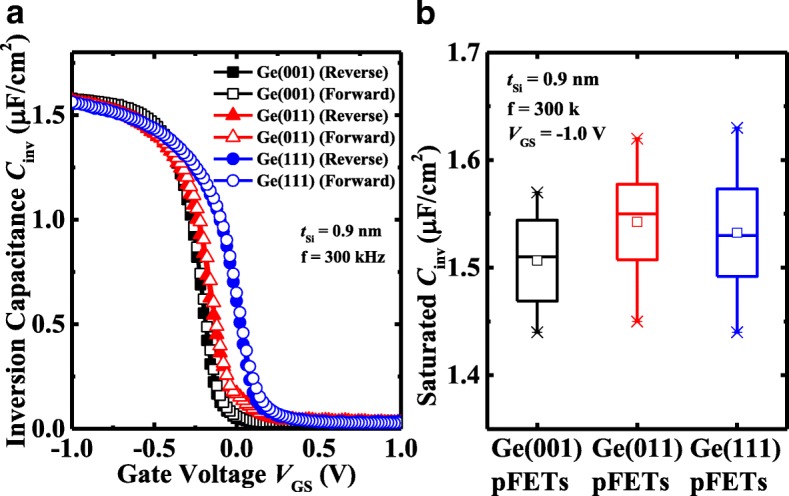
**a** Comparison of inversion *C*_inv_-*V*_GS_ curves among the Ge pMOSFETs with 0.9 nm *t*_Si_ on different orientations. Both forward and reverse sweeping are shown. **b** Statistical plots for the saturated *C*_inv_ of the devices showing the negligible differences in *C*_inv_ in the inversion regime

The thicknesses of Si/SiO_2_ IL in transistors with 0.9 nm *t*_Si_ on different surface orientations are studied by using inversion capacitance *C*_inv_ versus *V*_GS_ measurement, as shown in Fig. 5. Forward and reverse sweeping measurements exhibit the negligibly small hysteresis in the devices. The transistors exhibit the similar magnitude of *C*_inv_, ~ 1.56 μF/cm^2^, corresponding to the capacitive effective thickness (CET) of 2.2 nm. Figure 5b show the statistical results of saturated *C*_inv_ for the devices, which demonstrate the very small difference in *C*_inv_ in the transistors on different surface orientations. This indicates that the passivation rate of amorphous Si by magnetron sputtering is independent of the surface orientation. The rule of left-right shifts of the *C*_inv_-*V*_GS_ curves is well consistent with that of *V*_TH_ for the devices in Fig. 4d, which might be induced by the slightly different doping concentration in different orientation substrates.

**Fig. 6:**
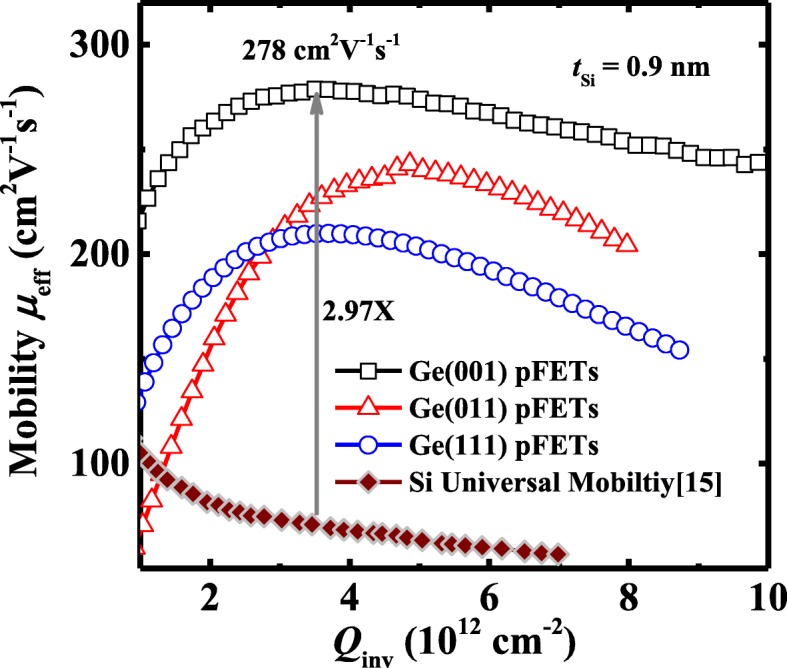
Plot of *μ*_eff_ versus *Q*_inv_ for Ge pMOSFETs with 0.9 nm *t*_Si_ on (001)-, (011)-, and (111)-oriented substrates. Ge(001) pMOSFETs achieve the 2.97 times enhancement in *μ*_eff_ at a *Q*_inv_ of 3.5 × 10^12^ cm^−2^ as compared to the Si universal mobility. The *μ*_eff_ was extracted using a total resistance slope-based method [17]

Figure 6 compares the mobility characteristics of the transistors with 0.9 nm *t*_Si_ on various surface orientations. The *μ*_eff_ was extracted using a total resistance slope-based method [14]. Ge(001) pMOSFETs exhibit the much higher channel mobility compared to the devices on (011) and (111) orientations. Transistors on (001) substrate achieve a peak *μ*_eff_ of 278 cm^2^/V·s at an inversion charge density *Q*_inv_ of ~ 3.5 × 10^12^ cm^−2^, which is 2.97 times higher than the Si universal mobility. Surface roughness at the Si/Ge interface and density of interface states (*D*_it_) can affect *μ*_eff_ of the devices at high inversion carrier density. It is unlikely that the commercially purchased Ge wafers with various surface orientations have the obvious difference in surface roughness. Therefore, it is speculated that the mobility enhancement in (001)-oriented devices is mainly due to reduced carrier scattering contributed by interface states. In this work, we evaluate the midgap *D*_it_ of the devices, and with the fixed *t*_Si_ of 0.9 nm, the (001)-oriented Ge pMOSFETs indeed have the lower midgap *D*_it_ compared to the other orientations.

**Fig. 7:**
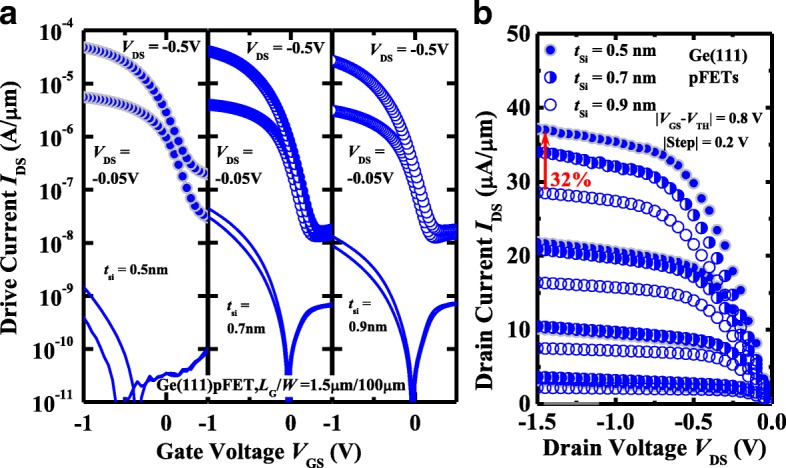
**a** *I*_DS_-*V*_GS_ and *I*_G_-*V*_GS_ and **b** *I*_DS_-*V*_DS_ curves of Ge(111) pMOSFETs with various *t*_Si_. Transistor with 0.5 nm *t*_Si_ exhibits a 32% improvement in *I*_ON_ compared to the device with 0.9 nm *t*_Si_ at *V*_DS_ of − 1.5 V and *V*_GS_-*V*_TH_ of − 0.8 V

**Fig. 8:**
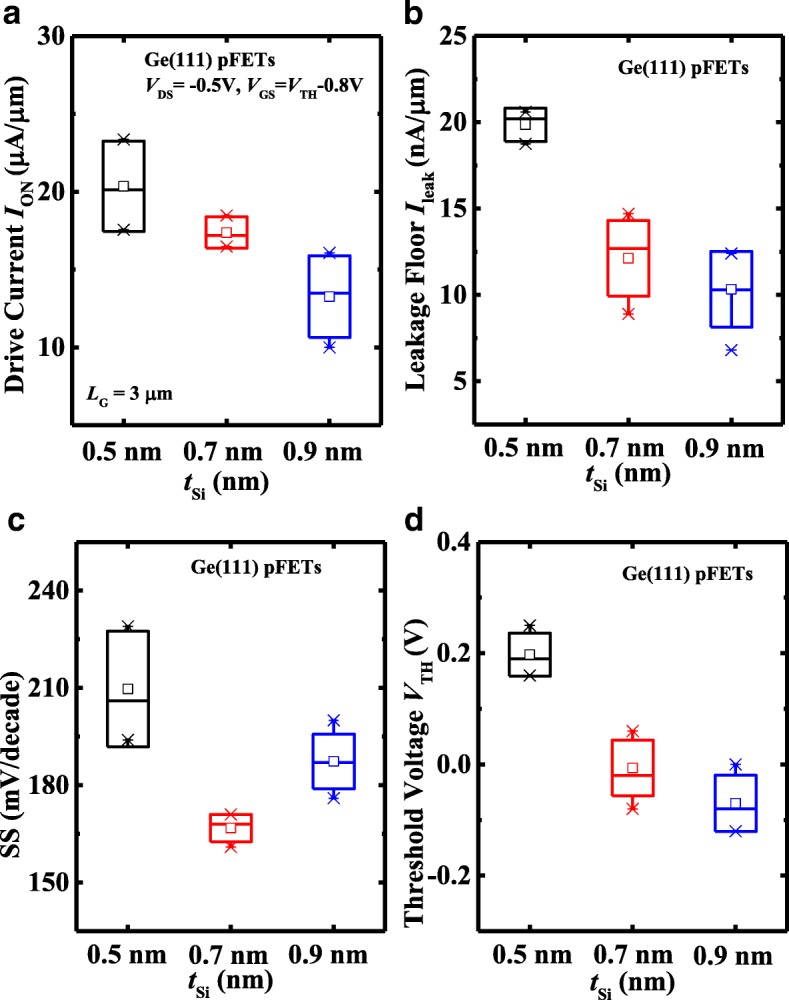
Comparison of **a** *I*_ON_, **b** *I*_leak_, **c** SS, and **d** *V*_TH_ for (111)-oriented Ge pMOSFETs with 0.5, 0.7, and 0.9 nm *t*_Si_ showing that transistors with 0.5 nm *t*_Si_ have the better *I*_ON_, but worse SS and *I*_leak_ characteristics in comparison with devices with thicker *t*_Si_

The impact of *t*_Si_ on the electrical performance of Ge pMOSFETs is also investigated. Figure 7a, b present the measured *I*_DS_-*V*_GS_ and *I*_DS_-*V*_DS_ curves, respectively, of the (111)-oriented Ge pMOSFETs with *t*_Si_ of 0.5, 0.7, and 0.9 nm at a *V*_DS_ of − 0.05 and − 0.5 V. The transistors have a *L*_G_ of 1.5 μm. It is observed that Ge pMOSFETs with 0.9 nm *t*_Si_ exhibit improved transfer characteristics compared to the devices with thinner *t*_Si_, but *I*_ON_ of the device decreases with the increasing of *t*_Si_. At *V*_DS_ of − 1.5 V and *V*_GS_-*V*_TH_ of − 0.8 V, Ge(111) pMOSFET with 0.5 nm *t*_Si_ demonstrates a 32% improvement in *I*_ON_ compared to the device with 0.9 nm *t*_Si_. Figure 8 plots the statistical results of *I*_ON_, *I*_leak_, SS, and *V*_TH_ of the Ge pMOSFETs on (111)-orientation with different *t*_Si_. From Fig. 8a, we see that transistors with 0.5 nm *t*_Si_ achieve the improved *I*_ON_ in comparison with the devices with thicker *t*_Si_, which is due to the transistor with 0.5 nm *t*_Si_ that has a smaller CET, leading to a higher *C*_inv_. It is noticed that *I*_leak_ decreases with the increasing of *t*_Si_ (Fig. 8b), and transistors with 0.5 nm *t*_Si_ has the inferior SS characteristics to those of the devices with 0.7 and 0.9 nm amorphous Si passivation layer (Fig. 8c). This might be due to those transistors with 0.5 nm *t*_Si_ having a higher midgap *D*_it_. The relation between SS and midgap *D*_it_ of Ge pMOSFET can be expressed by SS = ln(10) ⋅ (*kT*/q) ⋅ [1 + (*C*_it_ + *C*_*d*_)/*C*_*ox*_], where *C*_ox_, *C*_d_, and *C*_it_ are oxide capacitance, depletion-layer capacitance, and capacitance from interface traps, respectively. *C*_it_ can be calculated by *q* × *D*_it_, were *D*_it_ is the interface trap density. Although transistor with 0.5 nm *t*_Si_ has the larger *C*_ox_ compared to the other two devices, its higher midgap *D*_it_ can lead to the inferior SS to the devices with the thicker *t*_Si_. The surface passivation will also affect the *I*_leak_ from drain to source. With the sweeping of *V*_GS_ from position to negative, the channel transfers from accumulation mode to inversion mode. However, if the *D*_it_ is high, some points in channel surface are pinned by the interface traps, and the leakage paths can be formed, increasing *I*_leak_ from drain to source. As shown in Fig. 8d, Ge(111) pMOSFETs show the shift of *V*_TH_ to negative *V*_GS_ direction with the increasing of *t*_Si_, which is attributed to the increased CET. In addition, the density of traps in the lower bandgap half seems to increase for the thinner *t*_Si_, which might lead to the shift of *V*_TH_ [2].

**Fig. 9:**
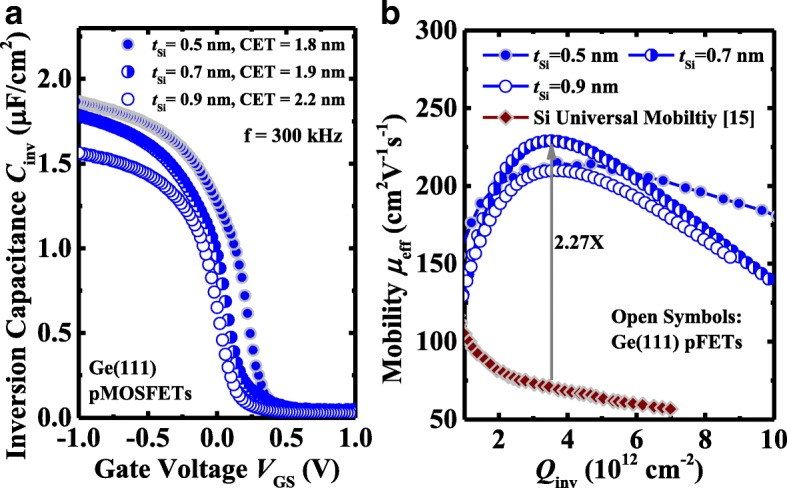
**a** *C*_inv_-*V*_G_ characteristics measured at 300 kHz for (111)-oriented devices with 0.5, 0.7, and 0.9 nm *t*_Si_. **b** *μ*_eff_ as a function of *Q*_inv_ for Ge pMOSFETs [17]

Figure 9a shows the *C*_inv_ as a function of *V*_GS_ curves for the Ge pMOSFETs on (111)-oriented surface with *t*_Si_ of 0.5, 0.7, and 0.9 nm measured at a frequency of 300 kHz. The CET values in inversion regions are extracted to be 1.8, 1.9, and 2.2 nm for the devices with 0.5, 0.7, and 0.9 nm *t*_si_, respectively. *μ*_eff_ as a function of *Q*_inv_ characteristics of the devices are extracted and shown in Fig. 9b. The (111)-oriented Ge pMOSFET with 0.7 nm *t*_si_ achieves the highest peak mobility of 229 cm^2^/V s, which is 2.27 times higher compared to the Si universal mobility. It should be noted that the devices with 0.5 nm *t*_Si_ exhibit a significantly improved *μ*_eff_ over the transistors with thicker *t*_Si_ at high *Q*_inv_ (e.g. 10^13^ cm^−2^). This also leads to the higher *I*_ON_ at high *V*_GS_-*V*_TH_ in the devices with 0.5 nm *t*_Si_ compared to the devices with 0.7 and 0.9 nm *t*_Si_. The *μ*_eff_ at high *Q*_inv_ decreases as *t*_Si_ increases from 0.5 nm to 0.7~0.9 nm, which is attributed to the fact that the larger surface roughness leads to the stronger surface roughness scattering of the carriers. During the passivation of Ge surface using magnetron sputtering at room temperature, the diffusion of surface atoms is greatly suppressed. So with the increasing of *t*_Si_, the surface roughness is larger, which can be observed from the HRTEM images in Fig. 2.

**Fig. 10:**
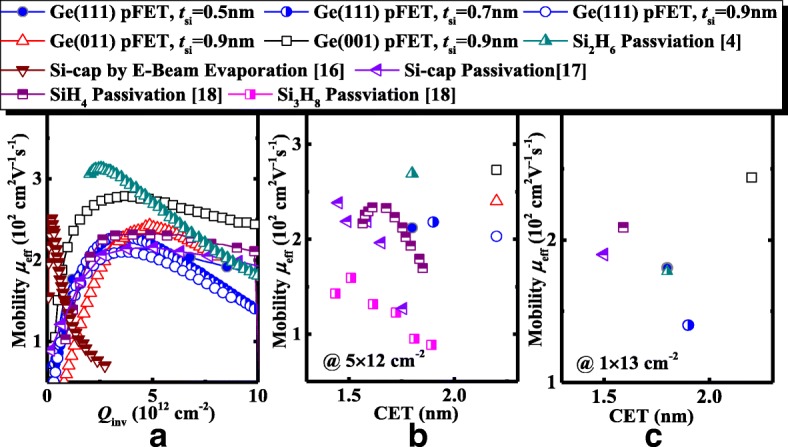
**a** *μ*_eff_ for the Ge pMOSFETs in this work vs. the published results for relaxed Ge pMOSFETs. **b**, **c** Benchmarking of *μ*_eff_ extracted at *Q*_inv_ = 5 × 10^12^ and 1 × 10^13^ cm^−2^, respectively, of the Ge pMOSFETs with the different CET values [18, 19]

In Fig. 10, we benchmark the *μ*_eff_ of the Ge pMOSFETs in this work with those of the reported relaxed Ge transistors with Si by E-beam evaporation, SiH_4_, Si_2_H_6,_ and Si_3_H_8_ passivation. Compared to the amorphous Si by E-beam evaporation in Ref. [15], Ge pMOSFETs in this work exhibit the significantly improved *μ*_eff_. It is seen that, at the similar CET, Ge pMOSFETs utilizing amorphous Si passivation by magnetron sputtering have the lower *μ*_eff_ in comparison with the devices with Si_2_H_6_ passivation. The process of passivation using amorphous Si needs to be further optimized to enhance the carrier mobility.

**Fig. 11:**
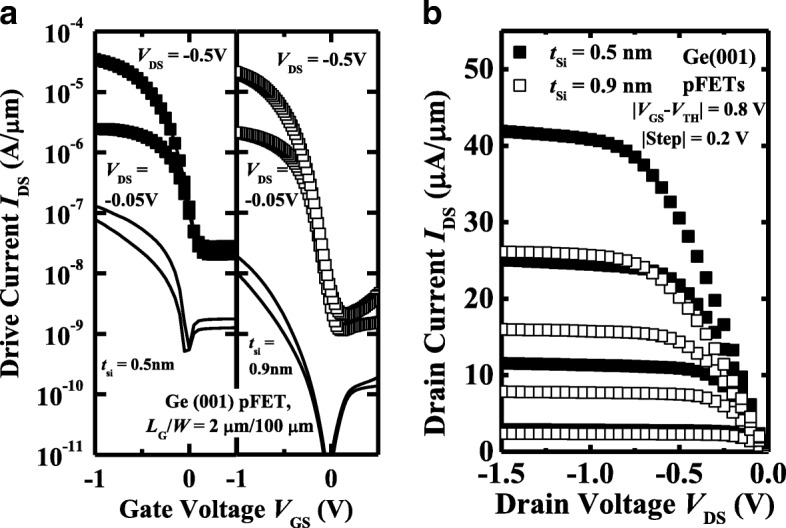
**a** Measured *I*_DS_-*V*_GS_ and *I*_G_-*V*_GS_ curves of (001)-oriented Ge pMOSFETs with 0.5 and 0.9 nm *t*_Si_. **b** *I*_DS_-*V*_GS_ curves of the devices

Ge pMOSFETs with the different *t*_Si_ on (001)-oriented surface are also characterized. Figure 11a, b illustrate the measured *I*_DS_-*V*_GS_ and *I*_DS_-*V*_DS_ curves, respectively, of a pair of Ge(001) pMOSFETs with 0.5 and 0.9 nm *t*_Si_. Similar to the (111)-oriented devices, Ge(001) pMOSFET with 0.5 nm *t*_Si_ obtains the improvement in *I*_ON_ but the degradation in *I*_leak_ compared to the transistor with 0.9 nm *t*_Si_.

**Fig. 12:**
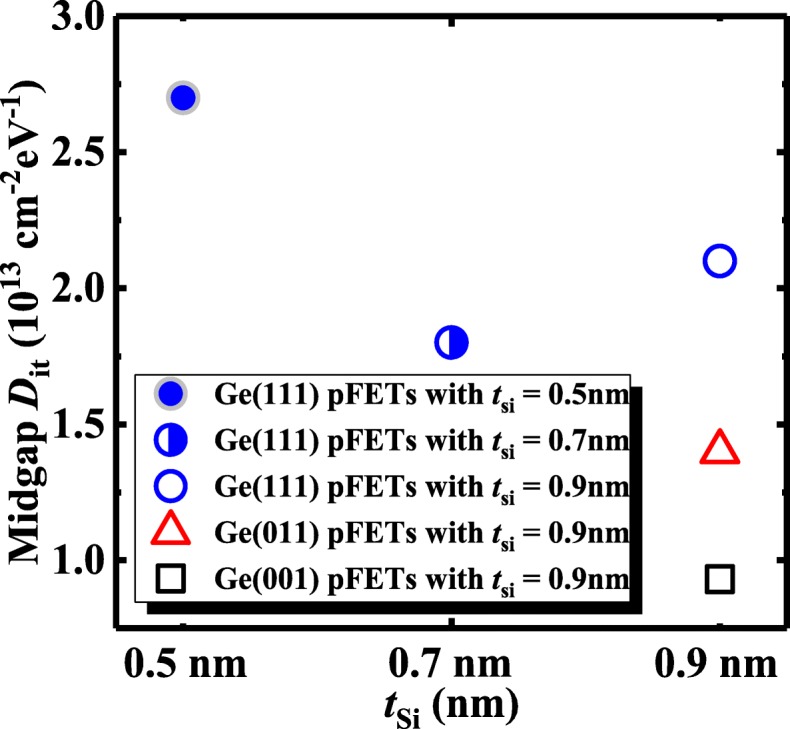
*D*_it_ versus the thickness of amorphous Si with various Ge surface orientations

The midgap *D*_it_ characteristics of Ge pMOSFETs are studied by the method in [16], and values of *D*_it_ are calculated by *D*_it_ = [SSlog(e)/(*kT*/*q*) − 1]*C*_G_/*q*, [16] where *q* is the electron charge, *k* is Boltzmann’s constant, *T* is the absolute temperature, and *C*_G_ is the measured gate capacitance per unit area. Figure 12 shows *D*_it_ as a function of the thickness of amorphous Si with various Ge surface orientations. For (111)-oriented surface, a device with 0.7-nm *t*_si_ has the lowest *D*_it_ value. With the 0.9 nm *t*_Si_, (001)-oriented device has the lower *D*_it_ compared to the transistors on other orientations.

Finally, we compare the key electrical characteristics of Ge pMOSFETs on the different orientations in Table 1. With a fixed *t*_Si_, Ge(001) pMOSFET has the improved electrical performance compared to the other two orientations. The drive current can be enhanced by reducing the *t*_Si_ from 0.9 nm to 0.5 nm, which is due to that the thinner *t*_Si_ provides a significantly reduced CET without causing degradation in *μ*_eff_.

**Table 1:** Key electrical performance of Ge pMOSFETs on the different orientations

Substrate orientation	*t*_Si_ (nm)	CET (nm)	Midgap *D*_it_ (cm^− 2^ eV ^− 1^)	SS (mV/decade)	*I*_ON_@*V*_DS_ = − 0.5 V, *V*_GS_-*V*_TH_ = − 0.8 V (*L*_G_ = 3 μm) (μA/μm)	*I*_leak_ (nA/μm)	*μ*_eff_@ *Q*_inv_ = 5 × 10^12^ cm^− 2^ (cm^2^/V × s)
(001)	0.9	2.2	9.3 × 10^12^	115	17.2	1.6	273
(011)	0.9	2.2	1.4 × 10^13^	159	15.1	5.5	240
(111)	0.9	2.2	2.1 × 10^13^	187	13.2	10.7	203
(111)	0.7	1.9	1.8 × 10^13^	166	17.3	12.3	218
(111)	0.5	1.8	2.7 × 10^13^	209	20.3	19.8	212

## Conclusions

Ge pMOSFET passivated by amorphous Si are demonstrated on (001)-, (011)-, and (111)-oriented substrate. With a *t*_Si_ of 0.9 nm, the improved *I*_ON_ and SS characteristics are obtained in (001)-oriented Ge pMOSFETs in comparison with the devices on (011) and (111) orientations, due to the higher *μ*_eff_ and lower midgap *D*_it_. Ge(001) pMOSFETs with 0.9 nm *t*_Si_ achieve a peak mobility of 278 cm^2^/V s at a *Q*_inv_ of 3.5 × 10^12^ cm^−2^, which is 2.97 times higher than the Si universal mobility. It is demonstrated that *I*_ON_ of the devices is improved with the decreasing of *t*_Si_ due to the reduction of CET. But Ge pMOSFETs with thicker *t*_Si_ exhibit the superior subthreshold swing and leakage floor, owing to that midgap *D*_it_ can be reduced by increasing *t*_Si_.

## Abbreviations

ALD: Atomic layer deposition
BF_2_^+^: Boron fluoride ion
CET: Capacitive effective thickness
Ge: Germanium
GeO_*x*_: Germanium oxide
HF: Hydrofluoric acid
HfO_2_: Hafnium dioxide
HRTEM: High-resolution transmission electron microscope
IL: Interfacial layer
MOSFETs: Metal-oxide-semiconductor field-effect transistors
Ni: Nickel
Si: Silicon
SS: Subthreshold swing
TaN: Tantalum nitride
TDMAHf: Tetrakis (dimethylamido) hafnium
